# Secondary malignancy risk for patients with localized prostate cancer after intensity‐modulated radiotherapy with and without flattening filter

**DOI:** 10.1002/acm2.13088

**Published:** 2020-11-04

**Authors:** Marius Treutwein, Rainer Loeschel, Matthias Hipp, Oliver Koelbl, Barbara Dobler

**Affiliations:** ^1^ Department for radiotherapy Regensburg University Medical Center Regensburg Germany; ^2^ Faculty of computer science and mathematics Ostbayerische Technische Hochschule Regensburg Germany; ^3^ Strahlentherapie Klinikum St. Marien Amberg Germany

**Keywords:** flattening filter free, IMRT, localized prostate cancer, secondary malignancy risk, VMAT

## Abstract

Men treated for localized prostate cancer by radiotherapy have often a remaining life span of 10 yr or more. Therefore, the risk for secondary malignancies should be taken into account. Plans for ten patients were evaluated which had been performed on an Oncentra® treatment planning system for a treatment with an Elekta Synergy™ linac with Agility™ head. The investigated techniques involved IMRT and VMTA with and without flattening filter. Different dose response models were applied for secondary carcinoma and sarcoma risk in the treated region and also in the periphery. As organs at risk we regarded for carcinoma risk urinary bladder, rectum, colon, esophagus, thyroid, and for sarcoma risk bone and soft tissue. The excess absolute risk (EAR) was found very similar in the treated region for both techniques (IMRT and VMAT) and also for both with and without flattening filter. The secondary sarcoma risk resulted about one magnitude smaller than the secondary carcinoma risk. The EAR to the peripheral organs was statistically significant reduced by application of the flattening filter free mode concerning the flattening filter as main source of scattered dose. Application of flattening filter free mode can thus support to reduce second malignancy risk for patients with localized prostate cancer.

## 1 INTRODUCTION

Prostate cancer is the most frequently diagnosed cancer among men in developed countries.^1^ Radiotherapy is a standard treatment modality with curative intent for localized prostate cancer. Although prostate cancer is a disease of elderly men, these patients have a remaining life span of 10 yr or more and therefore the risk for secondary malignancies should be taken into account. Radiotherapy compared to surgery may increase the risk for secondary cancer over time,^2^, ^3^, ^4^, ^5^ but there are also ambiguous results.^5^, ^6^, ^7^

Modern linear accelerators (linacs) promise shorter treatment times using the flattening filter free (FFF) mode. The flattening filter has been identified as the main source of scattered dose from the treatment head.^8^, ^9^ This dose might be responsible for additional secondary malignancy risk (SMR). Model calculations are regarded as a first essential step to evaluate this risk as long as clinical observations are not available.^10^ Only a few investigations using model calculations have been published about the impact of the FFF mode on SMR. Besides works about patients with breast cancer,^11^ ependymoma,^12^ and pituitary adenoma^13^ there is only one paper evaluating the SMR for patients with prostate cancer treated with linacs with and without flattening filter^14^ which confined to only three patients at cost of statistical significance. Additionally, they used another therapy planning system (TPS) which can affect the out‐of‐field dose.^15^

Minimizing the SMR can be one criterion in the decision for a specific technique apart from the plan quality. Therefore we compared the excess absolute risk (EAR) for secondary malignancies for the application of different fluence modulating treatment techniques, intensity modulated radiation therapy (IMRT) and volumetric modulated arc therapy (VMAT) with and without flattening filter.

## 2 MATERIALS AND METHODS

### 2.A Patients and regions of interest

Ten consecutive patients with histologically proven localized prostate cancer were included in this planning study. At the start of radiotherapy the patients had a mean age of 71 yr and all have given their written informed consent for participation in the planning study. The delineation of the regions of interest (ROI) in the TPS followed the description of Bos et al.^16^: The clinical target volume (CTV) was derived from the gross tumor volume (GTV) (prostate gland and seminal vesicals) by adding a 5 mm three‐dimensional margin excluding the rectal volume. Similarly, for the planning target volume (PTV) a margin of 10 mm was added to the GTV including parts of the rectum. The following organs at risk (OAR) were delineated: the rectal volume according to Guckenberger et al.,^17^ the urinary bladder, and the femoral heads. The bone structures were contoured automatically by standard bone window settings and corrected manually where it was appropriate. Soft tissue was delineated by subtracting bone and PTV from the outline contour.

### 2.B Linear accelerator

For the measurements and for the modeling in the TPS a linear accelerator type Elekta Synergy™ with Agility™ head (Elekta AB, Stockholm, Sweden) was applied. The head operates 80 interdigitating leaf pairs with a projected width of 5 mm at isocenter. It has been shown that the beam quality in flattened beam (FB) mode and FFF mode of 6 MV photons is very similar for energy matched Elekta machines^18^ what could be confirmed for the applied equipment.^19^ The maximum dose rates are 500 monitor units (MU) per minute for FB and 1700 MU per minute for FFF. The applied desktop software was Integrity R 3.2 and the record and verify system was Mosaiq 2.50.

### 2.C Treatment planning system

The TPS on which the optimizations were performed was Oncentra® External Beam v4.5 (Nucletron®, an Elekta AB) using the CC algorithm. Some publications have demonstrated the applicability of this system for treatments of prostate cancer and other entities with IMRT and VMAT and also for FB and FFF.^12^, ^28^ Limitations of the linear accelerator were considered setting the maximum of the variable gantry speed to a value of 6 degree per second and the minimum of the variable dose rate to a value of 20 MU per minute.

### 2.D Planning

The planning was set as simultaneous integrated boost in 33 fractions, aiming for 71.0 Gy minimum dose and 74.2 Gy maximum dose to the CTV which was used as boost volume. A minimum dose of 59.4 Gy was targeted for the PTV. For both modes FB and FFF, as well as both techniques IMRT and VMAT we applied the same set of dose volume objectives. The isocenter was localized to the center of the CTV.

The IMRT planning was performed to the study of Treutwein et al.^26^ with seven equispaced beams at gantry angles of 0°, 51°, 103°, 154°, 206°, 257°, and 309° and a collimator angle of 0°. The VMAT planning was optimized according to Treutwein et al.^27^ in a single arc gantry rotation from 182° to 178° and a collimator angle of 45°. Further planning details and investigations about the plan quality have been described in an earlier work.^28^

The dose grid spacing was set to 0.25 cm and the dose deposition was calculated to medium.

### 2.E Dose measurements

**Fig. 1:**
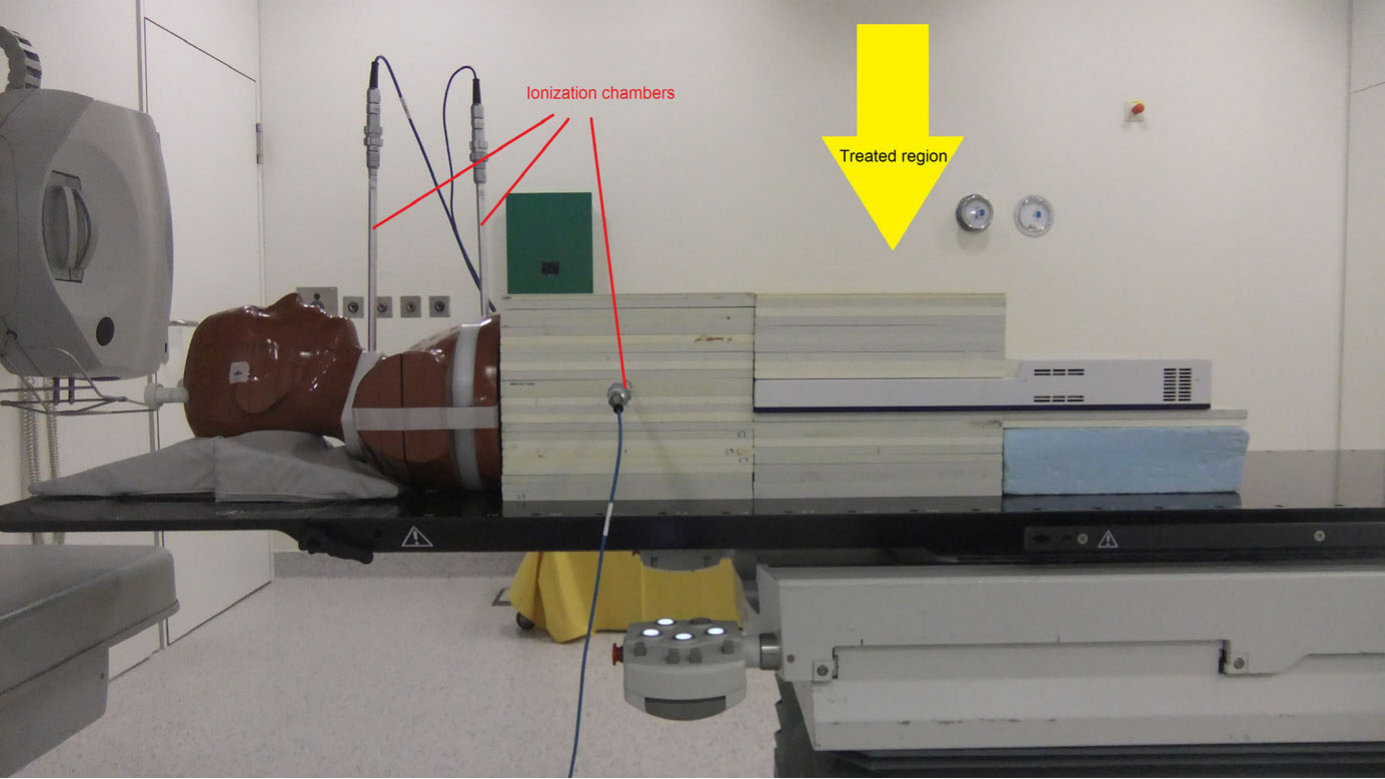
Setup of the phantom with inserted ionisation chambers in three positions.^27^

The measured dose to peripheral points was considered for the calculation of secondary malignancy risk in the periphery. Although these measurements have already been described in detail in the already mentioned work about investigations concerning plan quality ^28^, this is repeated here in short to facilitate the understanding. Two stacks of water equivalent material RW3 (PTW, Freiburg, Germany) were combined with the upper part of an Alderson phantom (RSD Inc., Long Beach, CA, USA) (Fig. 1). The caudal stack contained a 2D array for plan verifications which is not part of the present investigation. In the cranial stack one ionization chamber was introduced at a distance of 31 cm from the isocenter on the rotation axis of the gantry. This point corresponds approximately to the position of the transverse colon. To enable measurements in points corresponding to the lower esophagus and the thyroid gland, two slices of the Alderson phantom were replaced by PA material with bores for ionization chambers. All chambers were of type 30016 and the very similar 23332 (0.3 cm^3^ both) and connected to dosimeters of type Unidos (all of PTW Freiburg, Germany). These peripheral dose values will be labeled as PD^colon^, PD^esoph^, and PD^thyr^.

### 2.F Secondary malignancy risk

For the calculations of the secondary malignancy risk we used the models of Schneider et al.^29^ These models are based on a combination of the investigations of Preston et al.^30^ about the Japanese A‐bomb survivors and of Dores et al.^31^ about secondary cancer of Hodgkin’s patients after radiotherapy. We calculated the risk as excess absolute risk (EAR). It describes the absolute difference of the number of malignancies between a treated group and an untreated control group. It is expressed per 10.000 persons per year. The EAR can be factorized in functions of the sex *s*, the age at exposure *e*, the attained age *a*, and of the dose *d*.

(1) $$EAR\left(d,s,e,a\right)=\mu \left(s,e,a\right)\times f\left(d\right)$$

Preston et al. showed that for low dose regions up to a total dose *D* of about 2 Gy the linear dose response model is valid:

(2) $$EAR^{\mathrm{org}}=\beta_{\mathrm{EAR}}\times D\times \mu \left(e,a\right)$$

This linear dependence has also been assumed in a recent publication about 3D conformal radiation therapy of patients with prostate cancer.^32^ Preston gives values for different organs for the slope $\beta_{\mathrm{EAR}}$ which refer to the Japanese population. In some cases gender specific values are given. According Schneider et al. these Japanese values were corrected for western population (United Kingdom, UK) for selected organs^29^ (Table 1).

**Table 1:** Correction of the linear slope in the linear EAR model for western population.

Organ at risk	β_EAR_ (Japan)	Correction factor	β_EAR_ (UK)
Thyroid gland	0.5	0.35	0.2
Colon	13	0.92	12
Urinary bladder	3.8	1.2	4.6

The EAR for different organs of volume *V_T_* in the treated region was calculated with dose–volume data from the TPS by application of the tables given by Schneider et al.^29^:

(3) $$EAR^{\mathrm{org}}=\frac{1}{V_{T}}\sum_{i}V\left(D_{i}\right)\times \beta_{\mathrm{EAR}}\times RED\left(D_{i}\right)\times \mu \left(e,a\right)$$

$D_{i}$ is the dose in voxel i with volume *V*.$\beta_{\mathrm{EAR}}$ is the initial slope and the risk equivalent dose *RED* the dose dependent part. Factor *µ* is used to calculate the risk for different ages:

(4) $$\mu \left(e,a\right)=exp\left(\gamma_{e}\left(e‐30\right)+\gamma_{a}\ln\left(\frac{a}{70}\right)\right)$$

We used an age at radiotherapy of *e = 60* yr and an attained age of *a = 80* yr as proposed by Murray et al.^14^ The modifying parameters $\gamma_{e}$ and $\gamma_{a}$ for age correction have been taken from Preston et al.^30^ The values for these parameters and others which are explained hereafter are given in Table 2.

**Table 2:** Initial slope $\beta_{\mathrm{EAR}}$, α according the linear‐quadratic model and modifying parameters for the age correction.^29^, ^30^ The values below the double line refer to the sarcoma model.

OAR	$\beta_{\mathrm{EAR}}$	Mechanistic model	Mechanistic model	Bell‐shaped model (R = 0)	Plateau model (R = 1)α	$\gamma_{e}$	$\gamma_{a}$
		α	R	α			
Urinary bladder	4.6^a^	0.219	0.06	0.213	0.633	−0.024	2.38
Rectum	0.73	0.033	0.56	0.031	0.065	−0.056	6.9
Thyroid	0.2^a^	–	–	–	–	−0.046	0.6
Esophagus	3.2	–	–	–	–	–	–
Colon	12^a^	0.001	0.99	0.001	0.001	−0.056	6.9
Soft tissue	0.60	0.060	0.5			−0.013	−0.56
	3.30	0.040	0.1				
	0.35	0.093	1				
Bone	0.20	0.067	0.5			−0.013	−0.56
	1.7	0.019	0.1				
	0.10	0.010	1				

^a^ Gender specific values, valid for men only.

Schneider developed different models for carcinoma induction to determine the *RED*:

- The mechanistic model which considers cell killing and fractionation effects
- The bell‐shaped dose response model which neglects any repopulation or repair effect
- The plateau model with full repopulation or repair.

The mechanistic model is the most complex and considers fractionated treatment schedules with single fraction dose d up to a total dose D:

(5) $$RED\left(D\right)=\frac{e^{‐\alpha^{\prime }D}}{\alpha^{\prime }R}\left(1‐2R+R^{2}e^{\alpha^{\prime }D}‐{\left(1‐R\right)}^{2}e^{\frac{\alpha^{\prime }R}{1‐R}D}\right)$$

The parameter *α’* has been derived from the linear‐quadratic model:

(6) $$\alpha^{\prime }=\alpha +\beta d$$

Schneider et al.^29^ demonstrated that the model is robust in variations of *α/β*. Therefore, they assumed *α/β = 3 Gy* for all tissues. *R* is the repopulation and repair parameter. It equals 1 for full repopulation or repair and 0 for no repair. In the limit of *R* to 0 the formula can be simplified to the linear‐exponential or bell‐shaped model:

(7) $$RED\left(D\right)=De^{‐\alpha^{\prime }D}$$

The plateau model is achieved in the limit of *R* to 1:

(8) $$RED\left(D\right)=\left(1‐e^{‐\alpha^{\prime }D}\right)/\alpha^{\prime }$$

All three models were included in our investigation, as there is still little knowledge about the accurate shape of dose–response relationships for radiation induced cancer.^29^ Additionally the model for secondary sarcoma induction of bone and soft tissue was applied. The formula is quite similar to the mechanistic model for carcinoma induction, but contains an additional term:

(9) $$RED\left(D\right)=\frac{e^{‐\alpha^{\prime }D}}{\alpha^{\prime }R}\left(1‐2R+R^{2}e^{\alpha^{\prime }D}‐{\left(1‐R\right)}^{2}e^{\frac{\alpha^{\prime }R}{1‐R}D}‐\alpha^{\prime }RD\right)$$

Schneider et al.^29^ derived parameters for different repair and repopulation: Low repopulation (*R* = 0.1), intermediate repopulation (*R* = 0.5), and full recovery (*R* = 1.0).

### 2.G Statistics

We assumed as null hypothesis that the mean values are equal in both treatment modes FB and FFF.

The type I error should be smaller than 5% (*α* = 0.05). The Wilcoxon signed‐rank test for paired samples was selected as statistical test as the different plans were optimized on the identical sets of patient images. To control the maximum experimentwise error rate for multiple testing we applied the Bonferrroni–Holm correction.^33^ This correction considers the number *n* of evaluated variables or more specifically the corresponding null hypothesis. The *p* values for all *n* hypothesis are sorted in ascending order. Each hypothesis *n_m_* with 1 ≤ *m* ≤ *n* is discarded as long as:

(10) $$p_{m}\leq \frac{\alpha }{n+1‐m}=p_{m}^{*}$$

The higher the number *n* of statistically evaluated variables (null hypothesis), the smaller becomes $p_{1}^{*}$.and hence the chance decreases to find any *p* value smaller than this one. Therefore, two sums of variables only were considered in this process. However, they include all investigated *EARs*: The sum of all calculated EAR from the dose volume histograms $EAR_{\mathrm{sum}}^{\mathrm{plan}}$ and the sum of the EAR calculated from the PD measurements $EAR_{\mathrm{sum}}^{\mathrm{PD}}$.

## 3 RESULTS

**Fig. 2:**
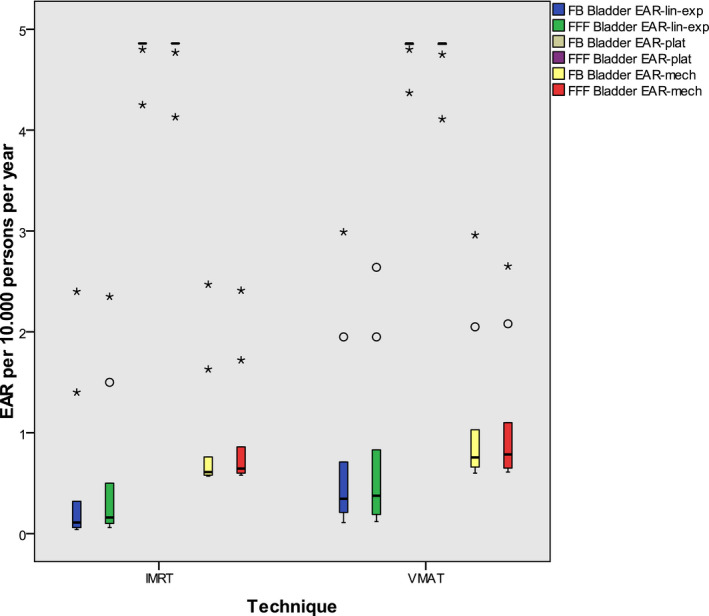
EAR for secondary bladder carcinoma including all plans: The boxes indicate the inner quartiles, the whiskers the outer quartiles; the boxes for the plateau model are horizontal lines only. Outliers and extreme values are indicated by circles and asterisks.

**Fig. 3:**
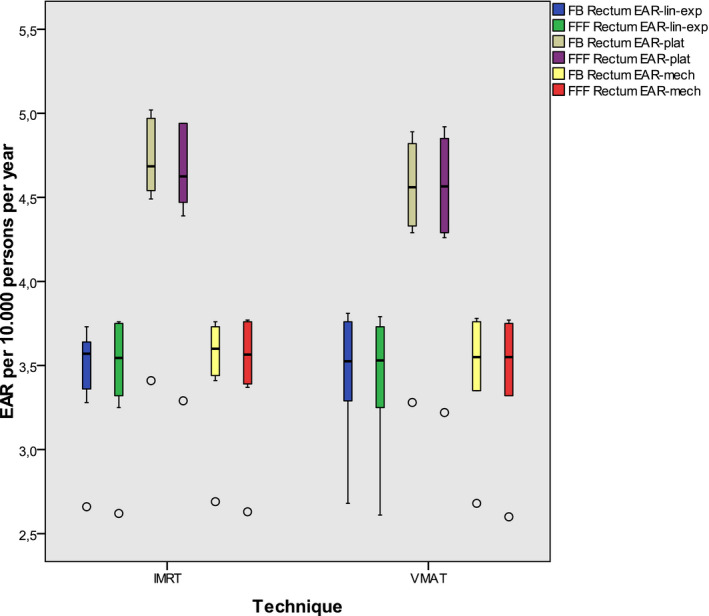
EAR for secondary rectum carcinoma including all plans: The boxes indicate the inner quartiles, the whiskers the outer quartiles. Outliers are indicated by circles.

The EAR for secondary cancer of the urinary bladder and the rectum for the selected age range depending on the risk model is demonstrated in Figs. 2 and 3. Only slight differences can be seen between IMRT and VMAT and also between FB and FFF.

**Fig. 4:**
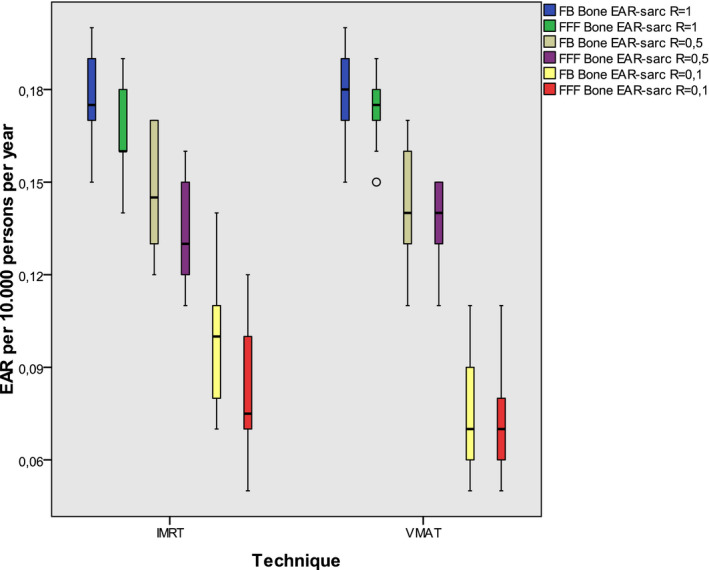
EAR for secondary bone sarcoma including all plans: The boxes indicate the inner quartiles, the whiskers the outer quartiles. Outliers are indicated by circles. R is the parameter for repair and repopulation and is represented by different colors.

**Fig. 5:**
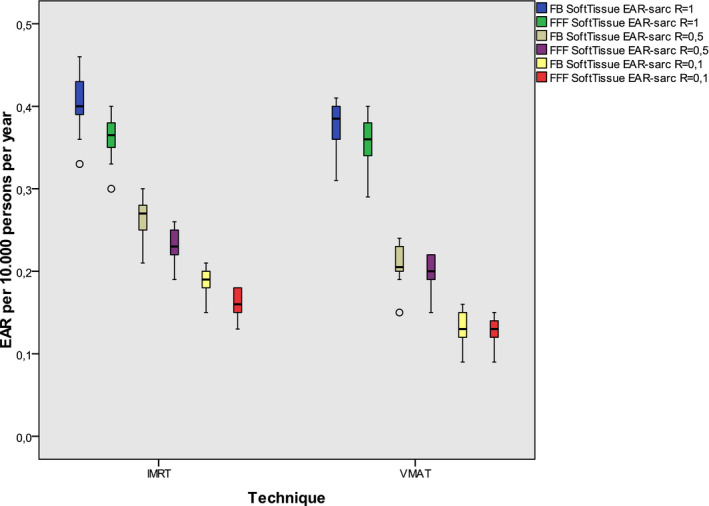
EAR for secondary soft‐tissue sarcoma including all plans: The boxes indicate the inner quartiles, the whiskers the outer quartiles. Outliers are indicated by circles. R is the parameter for repair and repopulation and is represented by different colors.

Box plots representing the EAR for secondary sarcoma risk of bone and soft tissue are shown in Figs. 4 and 5. Both figures show that the EAR is highest when the repopulation parameter *R* equals 1, that means full repopulation. It decreases when the repopulation is less complete. The risk for secondary sarcoma is about one order smaller than for secondary cancer.

As described in the previous paragraph about statistics only the sum of all EARs in the treated region was statistically evaluated. For each of the secondary carcinoma models one sum was calculated. For the sake of simplicity the sum for secondary sarcoma was included for the intermediate repopulation *R = 0.5* only. For the comparison of FB vs FFF no significance was found for the VMAT plans with p values between 0.13 and 0.28; for IMRT the difference between FB and FFF was found statistically significant for the plateau model only (*P* = 0.005); with p values of 0.08 (mechanistic model) and 0.61 (linear‐quadratic model) the $EAR_{\mathrm{sum}}^{\mathrm{plan}}$ was found equivalent for FB and FFF in the other models. Regarding the $EAR_{\mathrm{sum}}^{\mathrm{plan}}$ mean value and standard deviation for the plateau model numerically shows that the statistically significant difference is without clinical importance (FB: 9.8 ± 0.7; FFF: 9.7 ± 0.7).

**Fig. 6:**
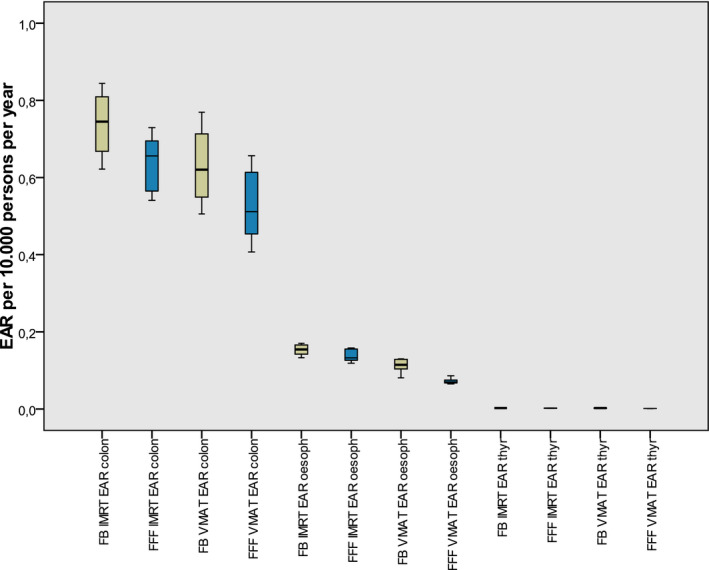
EAR in the periphery calculated from single point measurements. The boxes indicate the inner quartiles, the whiskers the outer quartiles. The three groups represent from left to right: colon, esophagus, and thyroid.

The EAR for the peripheral points was calculated using the linear model and the dose values from Table 3. The risk was very low compared to the secondary cancer risk in the treated region. Due to the high linearity factor $\beta_{\mathrm{EAR}}$ of the colon, this EAR has the major contribution as shown in Fig. 6.

**Table 3:** Peripheral dose in 3 points as shown in Fig. 1 for one fraction.^28^

Point	IMRT FB	IMRT FFF	VMAT FB	VMAT FFF
PD^colon^ in mGy	3.6 ± 0.4	3.0 ± 0.3	3.4 ± 0.4	2.5 ± 0.4
PD^esoph^ in mGy	1.5 ± 0.1	1.3 ± 0.1	1.1 ± 0.1	0.7 ± 0.1
PD^thyr^ in mGy	1.3 ± 0.1	1.1 ± 0.2	1.2 ± 0.1	0.6 ± 0.1

Again the sum $EAR_{\mathrm{sum}}^{\mathrm{PD}}$ was investigated statistically. For the FFF plans it was found significantly lower than for the FB plans (*P* = 0.005) at both techniques IMRT and VMAT. For IMRT the $EAR_{\mathrm{sum}}^{\mathrm{PD}}$ was reduced by 12%, for VMAT by 20%. Using VMAT reduced the risk compared to IMRT for 17% (FB) respectively 24% (FFF).

## 4 DISCUSSION

Schneider pointed out that even the combination of the two data sets of atomic bomb survivors and morbus Hodgkin patients involves uncertainties and problems.^29^ Additionally, differences in the three dose response models reveal a further range of possible values. Other procedures to calculate the second cancer risk from dose volume data would probably again end in different results as, for example, presented by Dasu et al. using cell survival in application of the linear‐quadratic model.^34^, ^35^ Therefore, our presented results must be regarded as a draft illustrating magnitude and relations of second cancer risk. In a narrow sense they are only valid for the described material and methods.

### 4.A Secondary malignancy risk in the treated volume

Schneider published for many investigated organs plots of the EAR in dependence of the dose.^29^ For the urinary bladder the plateau model is high above the other two models in the high dose region. Although these plots end at a maximum dose of 40 Gy, it seems reasonable that the results of the present study are similar, as the models are based on them.

In another work Schneider stated a slight increase in the secondary malignancy risk in the comparison of IMRT and 3D conformal technique in the treatment of prostate cancer,^36^ applying the bell‐shaped dose response model and the plateau model. This is in accordance with the results presented by Murray et al. in a review^37^: They demonstrated either none or slight increase only of secondary cancer after radiation for prostate cancer. However, the majority of the investigated 47 publications was at the basis of older techniques without intensity modulation. We calculated a maximum value EAR of about 5 per 10.000 men per year for both secondary urinary bladder and rectum carcinoma. This small number explains that differences are hardly detectable in clinical studies. Our results are of the same magnitude as Murray’s, calculated on three sample plans^14^ for patients with early prostate cancer.

Alvarez Moret et al. used the same dose response models in their investigation about SMR for the treatment of ependymoma. They stated that the difference between FB and FFF was statistically not significant for the application of IMRT, whereas for VMAT FFF the risk was significantly lower (2–3%) than for FB.^12^ Dobler et al. applied the models to plans for right‐sided breast cancer^11^ with and without flattening filter. They also described a dependency on the technique: for tangential arc VMAT the EAR at the contralateral breast and lung was significantly reduced with FFF, no differences were observed for VMAT, and for IMRT the EAR for the contralateral lung even increased with FFF. These examples illustrate that different localizations and techniques must be investigated separately, as such treatments depend on the patient, tumor location, and planning strategy.^15^

Brenner et al. evaluated second malignancies in patients with prostate cancer after radiotherapy.^2^ They compared them to a control group who underwent surgery only in a database analysis. The SMR was found significantly increased after radiotherapy. The documented risk increased to 1 radiation associated second malignancy in 70 patients who survived more than 10 yr. However, a detailed comparison is not possible due to different tumor staging, age, investigated time period, and treatment technique. Davis et al. also stated an increased risk for secondary rectum cancer after radiotherapy for prostate cancer but did not confirm it for urinary bladder^5^ whereas it was conversely stated by Neugut et al.^7^ Hegemann et al. doubt that increased rates of second cancer are caused by radiotherapy but assume lifestyle habits and comorbidities.^6^

The EAR for secondary sarcoma was found one magnitude smaller than for secondary cancer. This result is in accordance to the results of Preston et al. about the atomic bomb survivors.^30^ Although Schneider et al. concluded from data of radiotherapy patients that the risk might be of similar magnitude than carcinoma induction,^29^ this could not be confirmed for our given conditions. Also Brenner et al. documented an increased secondary sarcoma risk after radiotherapy which was smaller than the secondary carcinoma risk.

Cahan et al. reported in an early investigation that secondary bone sarcoma has rarely been observed after radiotherapy.^38^ Two recent studies about bone and soft tissue sarcoma after radiotherapy of breast cancer reported an increased risk after radiotherapy.^39^, ^40^ However, this would not override the benefit of radiotherapy. Virtanen et al. concluded that further investigation is necessary to resolve the dose response of the previous ionizing radiation.^41^

### 4.B Secondary malignancy risk in the periphery

For both techniques, IMRT and VMAT, the PD was smaller without than with flattening filter. As it has been mentioned in the introduction, the flattening filter has been described as a main source of scattered photons. However, the PD depends on many factors, as distance from the primary beam, the design of the treatment head including magnets and shielding material, and the treatment site.^8^, ^9^ The PD reduction in FFF mode was found common for different tumor localizations, linear accelerators, techniques, and TPS.^9^, ^11^, ^12^, ^14^, ^42^ It has been found the only general advantage of FFF in various normofractionated treatments.^43^

Similarly to Refs. [14, 42] we measured that the mean PD decreased with increasing distance from the field. However, this was not valid in every single case when comparing the dose to the thyroid and the esophagus point in individual plans. Different collimator configuration, head transmission dose, and scatter in the patient are supposed to be responsible.

VMAT compared to IMRT results in a lower PD. This has also been reported in some of the former mentioned publications.^12^, ^22^, ^28^, ^42^ It seems contradictory to the higher number of MU needed for VMAT in some of these reports — including our prostate cases. Obviously the respective collimator configuration plays an important role.

To our knowledge only in one study of Murray et al the SMR was calculated from the PD, applying the linear model as well.^14^ Our results were of the same magnitude regarding the esophagus and the thyroid. With FFF the risk was statistically significant reduced. As the SMR in the treated region is about one magnitude higher, the risk from the PD plays minor role in the decision process for a particular technique or mode.

## 5 CONCLUSIONS

The secondary malignancy risk in the radiotherapy treatment of patients with localized prostate cancer is very similar for both techniques (IMRT and VMAT) and both modes (FB and FFF) in the treated region. In peripheral regions it is statistically significant reduced for FFF on a low level. This can support the decision for a particular technique using FFF.
